# Effects of a stepwise, structured LDL-C lowering strategy in patients post-acute coronary syndrome

**DOI:** 10.1007/s12471-023-01851-7

**Published:** 2024-01-26

**Authors:** Aaram Omar Khader, Tinka van Trier, Sander van der Brug, An-ho Liem, Bjorn E. Groenemeijer, Astrid Schut, Harald T. Jorstad, Fabrice M.A.C. Martens, Marco A.M.W. Alings

**Affiliations:** 1 Amphia Hospital, Breda, The Netherlands; 2 Cardiovascular Institute, Thorax Center, Department of Cardiology, Erasmus MC, Rotterdam, The Netherlands; 3 Amsterdam University Medical Centers, Amsterdam, The Netherlands; 4 Franciscus Gasthuis, Rotterdam, The Netherlands; 5 WCN (Workgroup Cardiology Centres Netherlands), Utrecht, The Netherlands; 6 Gelre Hospitals Apeldoorn, Apeldoorn, The Netherlands; 7 Amsterdam Cardiovascular Sciences, Amsterdam Movement Sciences, Amsterdam, The Netherlands; 8 Deventer Hospital, Deventer, The Netherlands

**Keywords:** Cholesterol, LDL‑C, Hyperlipidemia/drug therapy, Hydroxymethylglutaryl-CoA reductase inhibitors, Ezetimibe, Proprotein convertase subtilisin/kexin type 9 inhibitors

## Abstract

**Objective:**

Low-density lipoprotein cholesterol (LDL-C) lowering constitutes a cornerstone of secondary prevention of atherosclerotic cardiovascular disease (ASCVD), yet a considerable number of patients do not achieve guideline-recommended LDL‑C targets. The 2016 European guidelines recommended titration of LDL‑C lowering medication in a set number of steps, starting with oral medication. We aimed to investigate the effects of this stepwise approach in post-acute coronary syndrome (ACS) patients.

**Methods:**

In a multicentre, prospective, non-randomised trial, we evaluated a three-step strategy aiming to reduce LDL‑C to ≤ 1.8 mmol/l in post-ACS patients with prior ASCVD and/or diabetes mellitus. Steps, undertaken every 4–6 weeks, included: 1) start high-intensity statin (HIST); 2) addition of ezetimibe; 3) addition of proprotein convertase subtilisin/kexin type 9 inhibitors (PCSK9i). The primary outcome was the proportion of patients achieving LDL-C ≤ 1.8 mmol/l after Steps 1 and 2 (using oral medications alone). Secondary outcomes examined the prevalence of meeting the target throughout all steps (https://onderzoekmetmensen.nl/nl/trial/21157).

**Results:**

Out of 999 patients, 84% (95% confidence intervals (CI): 81–86) achieved the LDL‑C target using only statin and/or ezetimibe. In an intention-to-treat analysis, the percentages of patients meeting the LDL‑C target after each step were 69% (95% CI: 67–72), 84% (95% CI: 81–86), and 87% (95% CI: 85–89), respectively. There were protocol deviations for 23, 38 and 23 patients at each respective step.

**Conclusion:**

Through stepwise intensification of lipid-lowering therapy, 84% of very high-risk post-ACS patients achieved an LDL‑C target of ≤ 1.8 mmol/l with oral medications alone. Addition of PCSK9i further increased this rate to 87% (95% CI: 85–89).

**Supplementary Information:**

The online version of this article (10.1007/s12471-023-01851-7) contains supplementary material, which is available to authorized users.

## What’s new?

- National and European guidelines recommend applying a stepwise approach to lower low-density lipoprotein cholesterol (LDL-C) to target, but low success rates have been observed in previous studies.
- Through a stepwise approach to cholesterol lowering, 84% of very high-risk cardiovascular patients reached target LDL‑C using only oral and affordable medication, increasing to 87% with additional use of PCSK9 inhibitors.
- A step-wise approach to LDL-lowering can be easily aligned with local considerations and implemented into clinical practice to improve and accelerate LDL‑C management in very high-risk patients.

## Introduction

Patients with atherosclerotic cardiovascular disease (ASCVD) and elevated low-density lipoprotein cholesterol (LDL-C) have a high residual cardiovascular risk [1]. International guidelines recommending strict LDL‑C targets are slowly being adopted in regional guidelines [2–6]. Statins are recommended as the first-choice medication to lower LDL‑C [2]. If LDL‑C targets are not met with high-intensity statin therapy (HIST), the addition of ezetimibe is indicated which may further reduce LDL‑C by up to 15–27% [4, 7]. Proprotein convertase subtilisin/kexin type 9 inhibitors (PCSK9i), used on top of statin therapy, reduce LDL‑C levels by an additional ~60% [8, 9]. Therefore, PCSK9i therapy is recommended for very high-risk patients in whom LDL‑C goals are not met despite a combination of maximum tolerated statin therapy and ezetimibe [2, 4–6]. While PCSK9i are highly effective in selected patients, they are not considered cost-effective for all ASCVD patients [10].

Despite guideline-based LDL‑C targets, there is a marked disparity with real-world clinical practice. Observational studies such as EUROASPIRE V and the DA VINCI study report that 29 and 54% of ASCVD patients, respectively, achieve LDL-C ≤ 1.8 mmol/l values. This proportion increases to 60% in a meta-analysis of statin trials [11–13]. These results underscore the challenges in effectively implementing lipid-lowering treatment, particularly considering the increasingly stringent LDL‑C guidelines in recent years.

We examined the impact of a protocolled three-step approach (1st HIST, 2nd HIST + ezetimibe, 3rd HIST + ezetimibe + PCSK9i) on achieving LDL‑C guideline-recommended targets in post-ACS patients, who are categorised as very high risk. While the 2019 and 2021 ESC Prevention and Dyslipidaemia guidelines advocate for a step-wise method to reach LDL targets, the practical implementation of such a strategy has not been explored in clinical practice [5, 6].

## Methods

### Design

The PENELOPE study was an investigator-initiated, multicentre, prospective, non-randomised trial, led and sponsored by the Workgroup Cardiology Centres Netherlands (WCN). The trial was conducted within 23 centres across the Netherlands. Financial support for the trial was provided by Sanofi. Per terms of the contractual agreement, Sanofi had no involvement in the design, execution, planning or publication of the trial.

### Study population

We enrolled patients > 18 years who were admitted for a type I ST-elevation myocardial infarction (STEMI) or non-ST-elevation myocardial infarction (NSTEMI), with a history of ASCVD and/or type 2 diabetes mellitus (T2DM). The inclusion criteria were designed to meet the definition of very high-risk ASCVD patients according to the 2016 ESC Guidelines and to fulfil the Dutch reimbursement criteria for PCSK9i (Tab. S1 of the Electronic Supplementary Material; [2, 14]). We excluded patients > 70 years with a Clinical Frailty Score > 3 or with a life expectancy < 1 year as preventive goals in these patients may differ, and patients not eligible for all steps in the protocol (pregnant or lactating women, known intolerance for alirocumab, already using PCSK9i) (Tab. 1) [15, 16].

**Table 1:** Inclusion and exclusion criteria

*Inclusion criteria*
Age > 18 years
Admission for type I (N)STEMI and
History of T2DM and/or ASCVD defined as:
– Cerebrovascular disease (TIA, cerebral infarction, amaurosis fugax, retinal infarction)
– Coronary artery disease (MI, ACS, coronary revascularisation: coronary angioplasty or CABG)
– Peripheral artery disease (symptomatic and documented obstruction of a distal extremity artery or surgical operation (percutaneous transluminal angioplasty, bypass or amputation)
*Exclusion criteria*
Any of the following criteria:
– Age > 70 years and a Clinical Frailty Score^A^ > 3
– Pregnant or lactating women
– Known intolerance for alirocumab
– Active PCSK9i therapy
– Life expectancy < 1 year

*T2DM* type 2 diabetes mellitus, *ASCVD* atherosclerotic cardiovascular disease, *TIA* transient ischaemic attack, *MI* myocardial infarction, *ACS* acute coronary syndrome, *CABG* coronary artery bypass graft, *PCSK9i* proprotein convertase subtilisin/kexin type 9 inhibitors

^A^To measure the frailty score, the Canadian Study of Health and Aging Clinical Frailty Scale was used [15]

### Intervention and outcomes

**Fig. 1:**
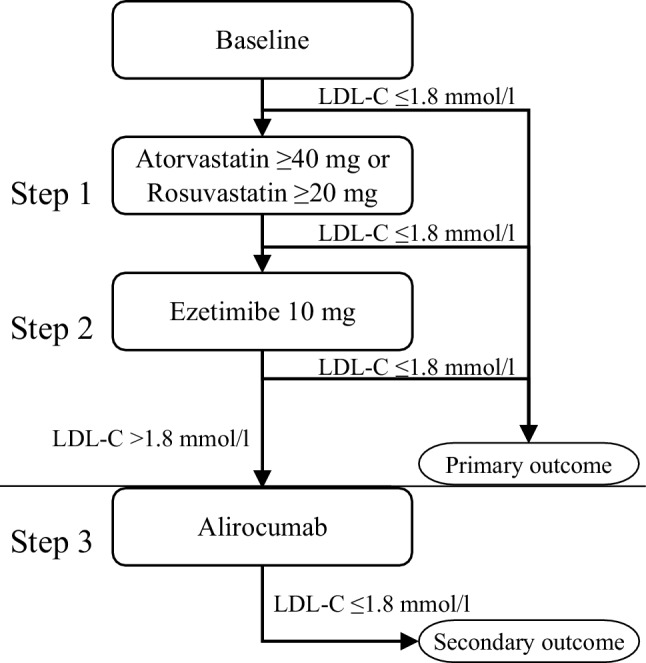
Study design flowchart. Step 1: Titrate/add high-intensity statin. If LDL-C > 1.8 mmol/l, move to Step 2: Add Ezetimibe. If LDL‑C remains > 1.8 mmol/l, proceed to Step 3: Add PCSK9i (dosing in Box S1). Typically, each step and subsequent LDL‑C check is 4–6 weeks apart, but in cases of statin intolerance, the gap between Steps 1 and 2 can extend to 12 weeks. *LDL‑C* low-density lipoprotein cholesterol

Patients underwent a structured, stepwise intensification process, comprising three consecutive steps: Step 1 adding/titrating to HIST, Step 2 adding ezetimibe and Step 3 adding PCSK9i (Fig. 1). After each step, every 4–6 weeks, lipid levels were measured and the next step would be initiated until the LDL‑C target (≤ 1.8 mmol/l) was reached. Therapy of patients on target, either at baseline or after any step, was not modified. Our primary outcome was the prevalence of patients reaching the LDL‑C target using oral medication only. Secondary outcomes included the prevalence of patients reaching target LDL‑C after each of the three steps.

#### Step 1: Starting or up-titrating HIST

Patients with an LDL‑C level > 1.8 mmol/l at baseline received HIST, atorvastatin (≥ 40 mg) or rosuvastatin (≥ 20 mg), or the highest tolerated statin dosage. A reduced dose or lower-intensity statin was prescribed when necessary due to factors such as low body weight or potential drug interactions. For patients with statin-related muscle symptoms, treatment adhered to the therapeutic guidelines set by the European Atherosclerosis Society Consensus Panel for managing statin-associated muscle symptoms [17].

#### Step 2: Adding ezetimibe

When LDL‑C persisted above 1.8 mmol/l despite HIST, ezetimibe 10 mg was introduced. Ezetimibe monotherapy was used for those intolerant to at least three distinct statins.

#### Step 3: Adding PCSK9i

If LDL‑C remained > 1.8 mmol/l after Steps 1 and 2, alirocumab could be added. The alirocumab dosing was left to the clinicians’ discretion which was based on age and LDL‑C level criteria (Electronic Supplementary Material, Box S1) including no alirocumab, or 75 mg or 150 mg every two weeks. This dosing strategy provided flexibility in treatment choices, particularly for lower LDL‑C, while ensuring effective therapy for higher LDL‑C levels. Lipid levels were assessed two weeks after the second alirocumab dose to determine secondary outcomes.

### Statistical methods

Baseline characteristics are presented using means with standard deviations (SD), medians with interquartile ranges (IQR), or percentages for categorical variables. Anticipating an 80% prevalence of LDL-C ≤ 1.8 mmol/l based on the IMPROVE-IT trial, a sample size of 1000 patients was calculated to achieve a 95% confidence interval (CI) with a 5% width for our primary objective (details in the Electronic Supplementary Material, Box S2; [7]). For each step, the percentage of patients attaining the LDL‑C target and the 5% CI are provided for both the entire cohort (intention-to-treat analysis) and those who rigorously followed the protocol (per-protocol analysis). Target achievement includes patients meeting the goal at the current step or in previous steps, including baseline. When the LDL‑C level was missing, non-high-density lipoprotein (non-HDL) < 2.6 mmol/l was used to determine target achievement. Patients missing both LDL‑C and non-HDL‑C levels were considered off-target in the intention-to-treat group and excluded from the per-protocol group. A post-hoc analysis evaluated patients achieving LDL-C ≤ 1.4 mmol/l, aligning with updated, stricter European guidelines despite not being the original study goal [6]. All analyses were performed in Python (version 3.10).

## Results

### Baseline characteristics

Between January 2019 and August 2020, 1000 patients were included, one of whom withdrew consent: 707 (70.7%) with NSTEMI and 292 (29.2%) with STEMI. Mean (SD) age was 67 (± 10) years, 23% were women, 74% had a history of ASCVD and 45% of T2DM. At baseline, 380 patients (38%) had LDL-C ≤ 1.8 mmol/l. Medication use at baseline is shown in Tab. 2. Among patients with established ASCVD, 85% were using any lipid-lowering treatment at baseline, and 43% were on target for LDL‑C. In patients with T2DM only, 50% were using any lipid-lowering treatment and 23% had LDL-C ≤ 1.8 mmol/l.

**Table 2:** Baseline characteristics

		LDL‑C mmol/l Median [Q1–Q3]	LDL-C ≤ 1.8 mmol/l^A^ *N* (%)
Total (*N*)	999	2.1 [1.5–2.8]	380 (38)
Age, mean, years (SD)	67 (± 10)		
≤ 70 years, *n* (%)	663 (66)	2.2 [1.6–3.0]	211 (32)
> 70 years, *n* (%)	336 (34)	1.8 [1.4–2.5]	169 (50)
*Sex, n (%)*			
– Female	229 (23)	2.2 [1.7–3.1]	70 (31)
– Male	770 (77)	2.01 [1.5–2.7]	310 (40)
*Lipid-lowering therapy, n (%)*			
– None	253 (25)	2.8 [2.0–3.7]	29 (11)
– HIST mono	228 (23)	1.8 [1.4–2.4]	120 (53)
– Other statins mono	387 (39)	1.91 [1.5–2.5]	170 (44)
– Ezetimibe mono	29 (3)	2.7 [2.4–3.2]	1 (3)
– HIST + ezetimibe	41 (4)	1.7 [1.4–2.2]	20 (49)
– Other statin + ezetimibe	61 (6)	1.6 [1.2–2.0]	40 (66)
*History, n (%)*			
Only T2DM	256 (26)	2.39 [1.9–3.1]	60 (23)
Only ASCVD^B^	544 (54)	2.1 [1.6–2.7]	211 (39)
ASCVD^B^ and T2DM	199 (20)	1.61[1.3–2.2]	109 (55)
Hypertension	592 (59)	2.0 [1.5–2.7]	242 (41)
Smoking	309 (31)	2.2 [1.6–2.9]	98 (32)
CKD (eGFR < 60 ml/min)	134 (13)	1.80 [1.4–2.6]	67 (50)

*T2DM* diabetes mellitus type 2, *ASCVD* atherosclerotic cardiovascular disease, *HIST* high-intensity statin therapy, *mono* monotherapy, *CKD* chronic kidney disease

^A^In case of missing baseline LDL‑C measurement, non-HDL < 2.6 mmol/l was used to determine the proportion of patients on target

^B^In total 743 patients have a history of ASCVD of whom 636 with a history of coronary artery disease, 129 with cerebrovascular disease and 86 with peripheral artery disease

The stepwise lipid-lowering treatment protocol was correctly applied in 915 (92%) patients (per-protocol) (See Box S3 and Fig. S1 in the Electronic Supplementary Material for detailed results per step).

### Primary outcome

**Fig. 2:**
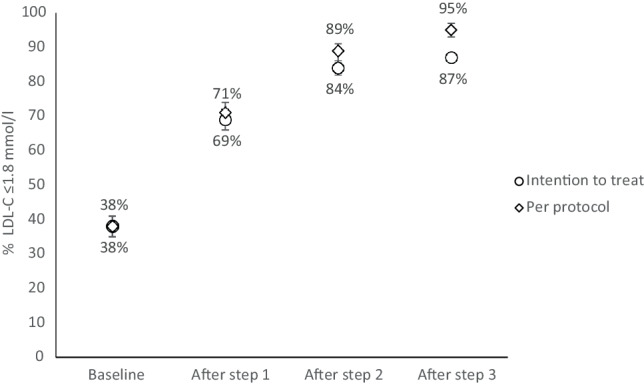
The prevalence of reaching LDL-C ≤ 1.8 mmol/l in the consecutive steps. Cumulative prevalence of patients achieving LDL-C ≤ 1.8 mmol/l from baseline through Step 1 (high-intensity statin), Step 2 (addition of ezetimibe—primary outcome), to Step 3 (addition of alirocumab). This figure visually depicts Tab. 3 data for the LDL‑C target of 1.8 mmol/l

The primary outcome was reached in 89% (CI: 87–91) of the patients in the per-protocol group, and 84% (CI: 81–86) in the intention-to-treat group. At baseline, 38% (CI: 35–41) of patients were on target. As a cumulative result of treatment with HIST (Step 1), HIST plus ezetimibe (Step 2) and HIST plus ezetimibe plus PCSK9i (Step 3), target LDL‑C was met in 71% (CI: 68–74), 89% (CI: 87–91) and 95% (CI: 94–96) in the per-protocol group (denominator at successive steps: *n* = 976, *n* = 938 and *n* = 915) and 69% (CI: 67–72), 84% (CI: 81–86) and 87% (CI: 85–89) in the intention-to-treat group (*n* = 999), respectively. The results (Tab. 3) are visualised in Fig. 2.

**Table 3:** Prevalence of reaching LDL-C ≤ 1.8 mmol/l and LDL-C ≤ 1.4 mmol/l in consecutive steps

Patients reaching target (*N*)			Intention to treat % (95% CI)	Per protocol % (95% CI)
	New	Total		
*LDL ≤* *1.8* *mmol/l*				
Baseline		380	38 (35–41)	38 (35–41)
Step 1: Adding HIST	+314	694	69 (67–72)	71 (68–74)
Step 2: Adding ezetimibe	+142	836	84 (81–86)	89 (87–91)
Step 3: Adding PCSK9i	+34	870	87 (85–89)	95 (94–96)
*LDL ≤* *1.4* *mmol/l*				
Baseline		187	19 (18–20)	
Step 1: Adding HIST	+151	338	34 (32–35)	N/A
Step 2: Adding ezetimibe	+64	402	40 (39–42)	N/A
Step 3: Adding PCSK9i	+24	426	43 (41–44)	N/A

Results for both intention-to-treat and per-protocol analysis, with the number of patients treated according protocol being 976, 938, 915 for steps 1, 2 and 3 respectively. Importantly, the medication regimen was tailored to achieve an LDL‑C level of ≤ 1.8 mmol/l. The study did not consider a target of ≤ 1.4 mmol/l within its protocol, this analysis was performed post-hoc to explore whether novel, strict targets have been met

*CI* confidence interval, *PCSK9i* proprotein convertase subtilisin/kexin type 9 inhibitors, *N/A* not applicable

#### Step 1: Starting or up-titrating HIST

Of the 618 patients with baseline LDL-C > 1.8 mmol/l, HIST was initiated or statin was titrated to a higher dose in 553 (89%). As a result of Step 1 an additional 314 patients reached target LDL‑C after a median (IQR) of 35 (29–45) days. A total of 694 patients had reached target LDL‑C after Step 1, corresponding with 71% (CI: 68–74) of the per-protocol group and 69% (CI: 67–72) of the intention-to-treat group (Tab. 3). Tab. S2 of the Electronic Supplementary Material shows the prescribed type and dosage of statins.

#### Step 2: Adding ezetimibe

After ezetimibe was added in 234 of 282 (83%) patients who had not reached target LDL‑C after Step 1, an additional 142 patients achieved target LDL‑C after a median (IQR) of 35 (29–46) days. Cumulatively, corresponding to the primary outcome, after Steps 1 + 2, 836 (84%, CI: 81–86) patients reached target LDL‑C (Tab. 3).

#### Step 3: Adding PCSK9i

**Fig. 3:**
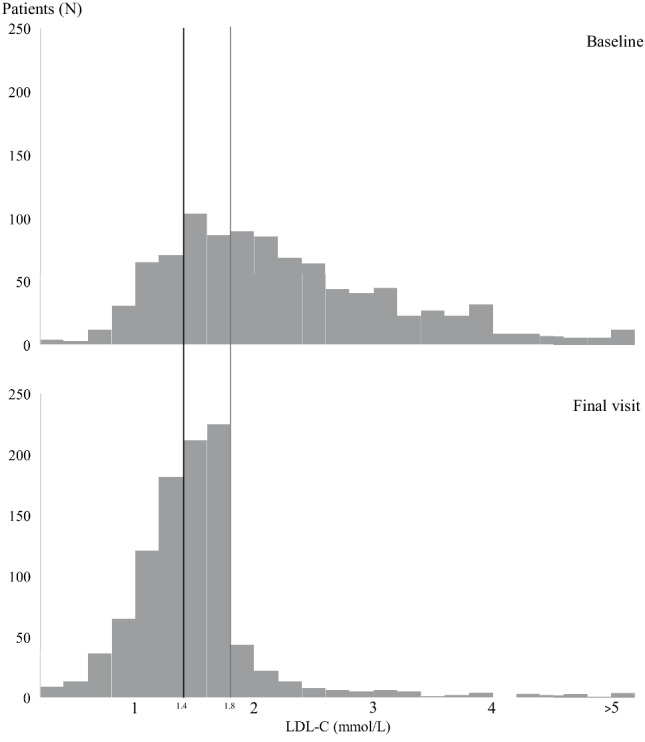
Low-density lipoprotein-cholesterol distribution at baseline and final visit. LDL‑C distribution at baseline and final visit. At the final visit: 4% had LDL-C > 2.6 mmol/l; 9% were between 1.8–2.6 mmol/L; 44% were between 1.4–1.8 mmol/l; and 43% achieved LDL-C ≤ 1.4 mmol/l. The treatment aimed for an LDL‑C level of ≤ 1.8 mmol/l

Patients remaining off-target after Steps 1 and 2 (*n* = 102) were eligible to receive PCSK9i. In 40 patients alirocumab was added on top of statin and/or ezetimibe, after which the target LDL‑C was met in an additional 34 patients. Prescribed dosages can be found in Box S1 in the Electronic Supplementary Material. In 39 patients with LDL-C ≤ 2.6 mmol/l PCSK9i therapy was not initiated because of the physician’s or patient’s preference. Overall, after completing Steps 1 + 2 + 3, target LDL‑C was reached in 870 patients, corresponding to 95% (CI: 94–96) of the per-protocol group and 87% (CI: 85–89) of the intention-to-treat group. The median (IQR) time to complete all three steps was 45 (32–77) days. The distribution of LDL‑C at baseline and after the final step is shown in Fig. 3. Even though medication adjustments were aimed at achieving an LDL-C ≤ 1.8 mmol/l, 43% of patients reached an LDL-C ≤ 1.4 mmol/l. The number of patients reaching LDL-C ≤ 1.4 mmol/l after each successive step is shown in Tab. 3.

### Protocol deviations

Overall, despite an LDL-C > 1.8 mmol/l, the protocol was not followed in 122 patients (12%): 22 patients were not prescribed HIST (Step 1), 38 patients were not prescribed ezetimibe (Step 2) and 62 patients were not prescribed PCSK9i (Step 3). The reasons for protocol deviation in the first two steps were study burden as perceived by the patient (*n* = 25), unknown reasons (*n* = 18), statin-related symptoms (*n* = 4), physician discretion (*n* = 4), deceased (*n* = 4), missing LDL‑C and non-HDL values (*n* = 1). The reasons for protocol deviation in Step 3 were not fulfilling reimbursement criteria due to ezetimibe intolerance in 7 patients; 16 patients with LDL-C ≥ 2.6 mmol/l did not receive PCSK9i due to patients’ and/or physicians’ choice. A detailed overview is shown in Tab. S3 of the Electronic Supplementary Material.

### Treatment intolerance

At baseline, 78 patients (7.8%) reported a history of statin intolerance; 55 of them had tried ≤ 2 statins before inclusion. Among those 55 patients, 51 (93%) tolerated a rechallenge with a third statin during Step 1 (HIST). Twenty-three patients with a documented history of intolerance to ≥ 3 statins were treated with ezetimibe instead of being rechallenged with statins. In the 553 patients without a history of statin intolerance, 46 (8%) patients developed muscle complaints, causing 12 patients (2%) to cease statin therapy. For ezetimibe, 12 (4%) patients reported side effects during the study.

## Discussion

This study evaluated a stepwise lipid-lowering strategy to achieve an LDL‑C of ≤ 1.8 mmol/l in post-ACS patients categorised as very high-risk patients. Utilising only statins and/or ezetimibe, 84% of the patients reached the target LDL‑C in two successive steps.

This stepwise approach combining statins, ezetimibe and PCSK9i led to better LDL‑C attainment rates than in most observational studies [11, 12, 18]. In line with the reigning 2016 ESC guideline at the beginning of this study, the LDL‑C target was set at ≤ 1.8 mmol/l. Although this still corresponds with current applicable Dutch cardiovascular guidelines and PCSK9i reimbursement criteria, the 2019 ESC guideline introduced a more stringent target of ≤ 1.4 mmol/l. Although medication adjustments were not aimed at the latter target, an LDL-C ≤ 1.4 mmol/l was achieved in 43% of the patients (Fig. 3 and Tab. 3). This demonstrates that the stepwise approach is adaptable to varied local thresholds and suggests it could be superior to commonly used titration strategies that depend on the healthcare professional’s discretion.

Implementing stepwise lipid-lowering treatment protocols requires a robust infrastructure due to the frequent visits and associated workload (Electronic Supplementary Material, Box S3). Yet, 84% of patients achieved LDL‑C targets exclusively using oral therapeutics, underscoring their affordability. Prioritising appropriate and early prescription of oral lipid-lowering treatment in such protocols may improve the accessibility and affordability of post-ACS care.

In this study, a low incidence of statin- and ezetimibe-related complaints was observed, even among patients with a prior history of statin-related issues. Re-challenging such patients might enhance the likelihood of achieving LDL targets with oral medications. These observations resonate with the findings from both the SAMSON and StatinWISE trials, which reported no significant differences in muscle-related symptoms between the statin and placebo groups [19, 20].

### Limitations

Some aspects of our study warrant consideration. First, during the course of the study, the 2019 ESC guidelines changed the LDL‑C target for very high-risk ASCVD patients from < 1.8 mmol/l to < 1.4 mmol/l or even ≤ 1.0 mmol/l. This target, however, is not yet adopted by many national societies and multidisciplinary guidelines, such as the Dutch multidisciplinary cardiovascular risk management guidelines [4]. Therefore, our study target of ≤ 1.8 mmol/l remains relevant [2]. Secondly, our study design purposefully permitted clinicians to decide on HIST and alirocumab dosages. While 623 patients (62%) were on HIST at their final visit, a mere 119 (12%) were on the maximum dosage of atorvastatin (80 mg) or rosuvastatin (40 mg) (Electronic Supplementary Material, Tab. S2). Moreover, of the 10% of the patients with an indication for PCSK9i, only 60% were given this treatment (Electronic Supplementary Material, Box S3). Adjusting to the top-end dosage of statins and ensuring complete PCSK9i coverage for all qualified patients might have enabled more patients to reach their LDL‑C goals. Restraint in prescribing this potent, newer class of lipid-lowering treatment emphasises the need for further education on the benefits, costs and potential risks, especially in this highly vulnerable patient subgroup. Finally, this study lacked a (randomly selected) control group, highlighting the need for future studies comparing protocolled, stepwise lipid-lowering treatment to usual care.

## Conclusion

A stepwise lipid-lowering strategy utilising only statins and/or ezetimibe resulted in 84% (95% CI: 81–86) of post-ACS patients—classified as very high-risk—achieving an LDL‑C level of ≤ 1.8 mmol/l. This affordable and simple approach has the potential to enable more patients to reach guideline-based lipid targets.

### Supplementary Information

Supplementary data

Statistical analysis plan

## Data Availability

The data underlying this article will be shared on reasonable request to the corresponding author.
