# Supplementary material for: Effects of a stepwise, structured LDL-C lowering strategy in patients post-acute coronary syndrome
Source: Neth Heart J. 2024 Jan 26;32(5):206–12. doi: 10.1007/s12471-023-01851-7 (PMC11039599; doi:10.1007/s12471-023-01851-7)
Supplement: Supplementary file 1 — Supplementary data [file 12471_2023_1851_MOESM1_ESM.docx]

**Content of Supplements**

PENELOPE study: Effects of a stepwise, structured LDL-C lowering strategy in patients post-acute coronary syndrome

**Table of content**

[**Table S1 Definitions of very high-risk patients 2**](#_Toc147391719)

[**Box S1 Dosing Schedule for Alirocumab: Protocol vs. Practice 3**](#_Toc147391720)

[**Box S2 Sample size calculation 4**](#_Toc147391721)

[**Box S3 Protocol in practice: results and implementation per step 5**](#_Toc147391722)

[**Fig. S1 Number of patients on and off LDL-target after each protocolled step 6**](#_Toc147391723)

[**Table S2 Actual prescribed statins at final visit 7**](#_Toc147391724)

[**Table S3 Protocol deviations with corresponding numbers and reasons 8**](#_Toc147391725)

[**References for supplemental material 9**](#_Toc147391726)

# Table S1 Definitions of very high-risk patients

| **ESC definition of very high-risk patients** [1] | **Dutch reimbursement criteria for PCSK9i** [2] | **PENELOPE study population** |
| --- | --- | --- |
| any of the following | any of the following | all of the following |
|  |  |  |
| - Documented CVD clinical or unequivocal on imaging.* | - T2DM and a CV event | - History of ASCVD and/or T2DM |
| - Severe CKD (GFR <30 mL/min/1.73) m2) | - recurrent CV event | - New type I (N)STEMI. |
| - A calculated SCORE ≥10%. | - Heterozygous FH |  |
| - DM with: | - CV event and documented statin-intolerance |  |
| - - Target organ damage or |  |  |
| - - with a major risk factors: - risk factors |  |  |
| - Smoking |  |  |
| - Hypertension |  |  |
| - Dyslipidaemia |  |  |

*Documented CVD includes previous acute coronary syndrome (myocardial infarction or unstable angina), stable angina, coronary revascularization (PCI, CABG, and other arterial revascularization procedures), stroke and transient ischaemic attack, and peripheral arterial disease. Unequivocally documented CVD on imaging is what has been shown to be strongly predisposed to clinical events, such as significant plaque on coronary angiography or carotid ultrasound. Abbreviations: CVD, cardiovascular disease; CKD, chronic kidney disease; ASCVD, atherosclerotic cardiovascular disease; FH, familial hypercholesterolemia; DM, Diabetes mellitus; CV, cardiovascular event; T2DM, Diabetes mellitus type II; (N)STEMI, (Non) ST segment elevation myocardial infarct.

# Box S1 Dosing Schedule for Alirocumab: Protocol vs. Practice

| Per Protocol Instructions | | |
| --- | --- | --- |
| **Age group** | **LDL-C (mmol/L)** | **Dosage options alirocumab (at investigator’s discretion)** |
| 18-70 | >1.8 - <2.6 | no alirocumab, alirocumab 75 mg Q2W or alirocumab 150 mg Q2W |
| 18-70 | ≥2.6 - <3.6 | alirocumab 75 mg Q2W or alirocumab 150 mg Q2W |
| 18-70 | ≥3.6 | alirocumab 150 mg Q2W |
| >70 | >1.8 | no alirocumab, alirocumab 75 mg Q2W or alirocumab 150 mg Q2W |
| Actual Practice | | |
| 18-70 | >1.8 - <2.6 | 30 patients no Alirocumab, 16 patients: alirocumab 75 mg Q2W and 1 patient Evolocumab 140mg |
| 18-70 | ≥2.6 - <3.6 | 9 patients alirocumab 75 mg Q2W, 4 patients alirocumab 150 mg Q2W, 1 patient Evolocumab |
| 18-70 | ≥3.6 | 4 patients alirocumab 150 mg Q2W |
| >70 | >1.8 | 9 patients no Alirocumab, 5 patients Alirocumab 75 mg |

This box presents the dosing regimen as directed by the protocol alongside the actual prescription given during the study. The protocol's alirocumab dosing provided investigators with treatment flexibility, particularly for lower LDL-C levels, while maintaining effective therapy for higher LDL-C values. Patients for whom the protocol wasn't followed are excluded from this box but can be found in Table S3.

**Box S2 Sample size calculation**

Sample size calculation:

The sample size was calculated to provide a prevalence (LDL ≤1.8 mmol/L in patients mentioned in the primary objective) with 95% confidence interval with a width (upper bound minus lower bound) of 5%.
The prevalence of LDL-C ≤1.8 mmol/L in patients treated with high-intensity statin and ezetimibe (~ primary objective) was estimated at 0.80 based on the IMPROVE-IT trial. [3] Seventy-five percent of patients on simvastatin and ezetimibe in the IMPROVE-IT trial have a LDL <62 mg/dL (1.6 mmol/L). Since a higher threshold (1.8 mmol/L) and more potent statins are used in the current study, the prevalence was estimated at 80%. Using this assumption, 983 patients are needed to ensure a 95% confidence interval with a width of 5% (margin of error: 2.5%). The number was rounded up to 1,000 patients.


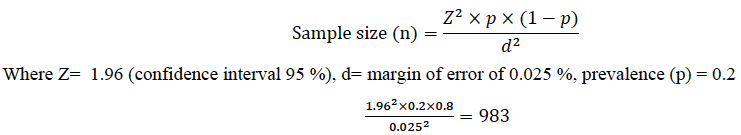


**Box S3 Protocol in practice: results and implementation per step**

*Baseline*

At baseline, 380 of 999 eligible patients were already on target, 1 patient was excluded as LDL-C was missing. The remaining 618 patients were suitable for Step 1 (HIST).

*Step 1*

Of 618 patients being eligible for step 1, 22 were excluded due to protocol deviations (table S3). Of the remaining 596 patients, 42 patients did not receive HIST according to the protocol (23 due to known statin intolerance; 19 due to baseline use of HIST plus ezetimibe), and in 1 patient HIST was not initiated in deviation from protocol. These 43 patients, however, remained in study follow-up and available for LLT other than HIST or ezetimibe. After step 1, an additional 314 reached the target (net effect), while the remaining 282 patients were eligible for step 2.

*Step 2*

Of 282 eligible patients in step 2, 38 were excluded due to protocol deviations (table S3). 244 patients in step 2, 10 already used ezetimibe at baseline and proceeded to step 3. After step 2, an additional 142 reached the target (net effect), while the remaining 102 patients were eligible for step 3, from which 23 patients were excluded due to various reasons (table S3).

*Step 3*

Of 102 patients being eligible to receive PCSK9i, 23 were excluded due to various reasons (table S3). Of 79 patients in step 3, 39 patients with LDL-C 1.8-2.6 mmol/L did not receive PCSK9i at physician’s discretion. This is not defined as protocol deviations, since our protocol left the decision to not initiate PCSK9i in these patients to the physicians and patients choice. The remaining 40 patients were treated with PCSK9i of which 34 reached the target LDL-C.

**Time-window**

The median (IQR) number of days, from baseline, needed to reach the target LDL-C was 35 (29-45), 42 (32-70) and 45 (32-77) respectively for step 1, step 2 and step 3. The short median time to complete all 3 steps reflects the fact that many patients had only statin intensification, without addition of ezetimibe or PCSKC9i.

The median (IQR) number of days between treatment modification at each consecutive step and LDL-C measurement to assess the effects were 35 (29-45) for step 1, 35 (29-46) for step 2 and 52 (38-69) for step 3.

A total of 1921 visits were required to achieve the aforementioned results.

**Missing LDL-C values:**

In total, 40 LDL-C values were missing. For 39 of these missing values, a corresponding non-HDL measurement was available, broken down as follows: baseline (27), step 1 (5), step 2 (3), and step 3 (4). One patient, with both LDL-C and non-HDL missing at baseline, was considered a protocol deviation.

**
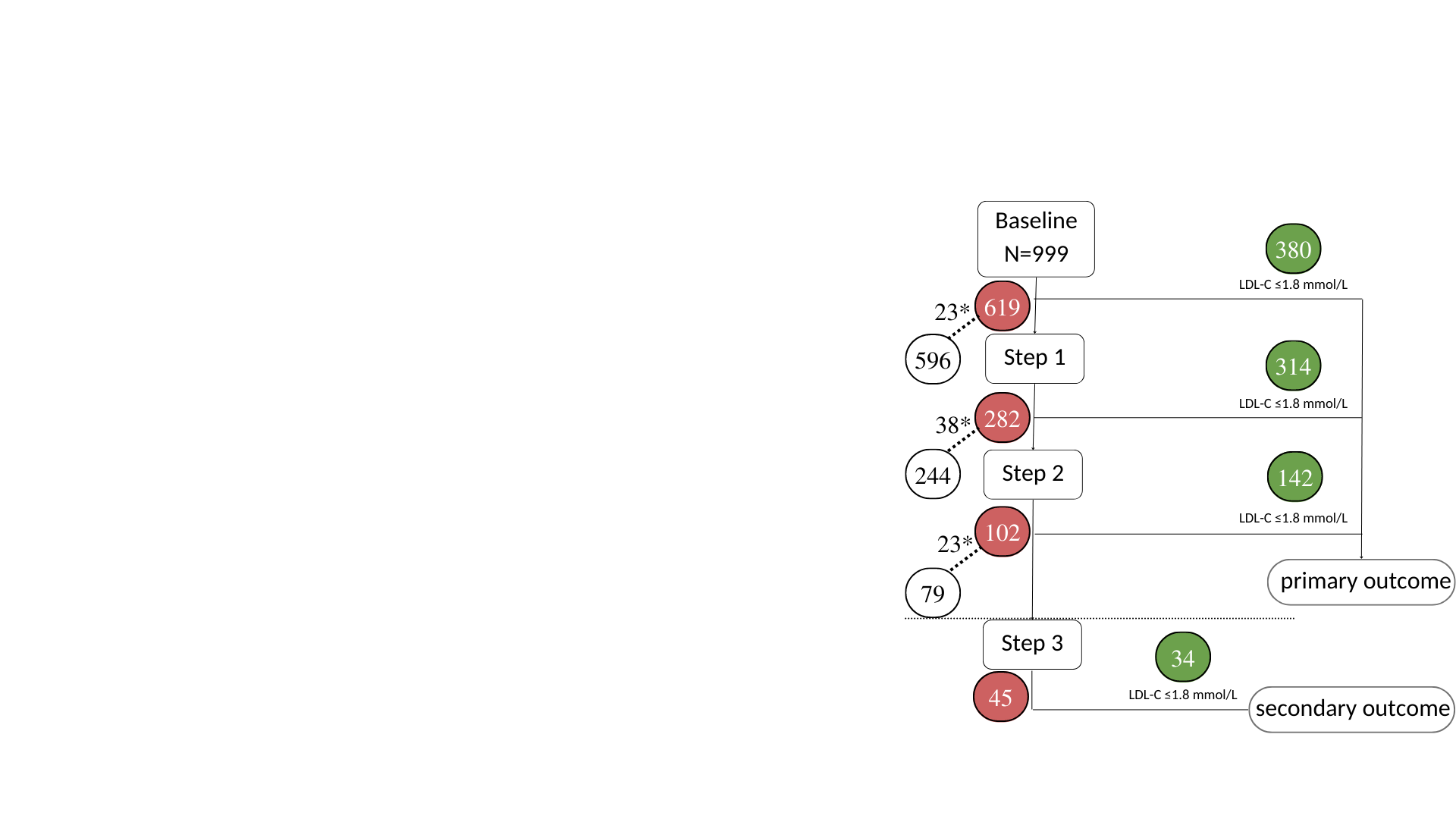
**

**Fig. S1 Number of patients on and off LDL-target after each protocolled step**

After each step, patients meeting the LDL-target are shown in green, while those off-target are in red. Off-target patients eligible to proceed to the next step. The asterisk (*) denotes the number of patients with protocol deviations, who didn't receive the subsequent protocolled treatment (see Table S3 for reasons). The white circle indicates the number of patients who actually proceeded to the subsequent step

# Table S2 Actual prescribed statins at final visit

| **Statin with dosage (mg/day)** | **Intensity** | **N** |
| --- | --- | --- |
| **None** |  |  |
| Baseline LDL-C≤ 1.8mmol/L | | 30 |
| Due to intolerance |  | 35 |
| Unknown reason |  | 10 |
| **Atorvastatin** |  |  |
| 10 | M | 17 |
| 20 | M | 28 |
| 40 | H | 380 |
| 80 | H | 93 |
| **Pravastatin** |  |  |
| 20 | L | 5 |
| 40 | M | 16 |
| Unknown dose |  | 1 |
| **Rosuvastatin** |  |  |
| 5 | M | 32 |
| 10 | M | 61 |
| 20 | H | 128 |
| 40 | H | 26 |
| Unknown dose |  | 1 |
| **Simvastatin** |  |  |
| 20 | M | 29 |
| 40 | M | 96 |
| 80 | - | 2 |
| Unknown dose |  | 1 |
| **Other statins** |  | 8 |
| **Total** |  | **999** |

At final study visit, 92% used any statin and 627 (63%) used high-intensity statin (ACC/AHA classification of intensity [4]). There is no official classification for simvastatin 80mg. Abbreviations: L, low; M, moderate; H, high.

**Table S3 Protocol deviations with corresponding numbers and reasons**

|  | **Protocol deviations, N** | **Reasons for protocol deviation** | **Denominator of per protocol group, N** |
| --- | --- | --- | --- |
| **Baseline (N=999)** | 1 | 1 missing LDL-C and non-HDL-C level | 998 |
| **Step 1** | 22 | 9 drop-out (study burden)  5 unknown reason  4 died  2 statin-related symptoms  2 physician’s discretion | 976 |
| **Step 2** | 38 | 16 drop-out (study burden)  13 unknown reason  5 medication non-adherence  2 statin-related symptoms  2 physician’s discretion | 938 |
| **Step 3** | 23 | 7 no reimbursement ^a^  16 with LDL-C≥2.6 mmol/L due to patients and/or physicians choice | 915 |

No further lipid-lowering therapy was adjusted in this group, despite an LDL-C >1.8 mmol/L. a.)Due to ezetimibe intolerance, 7 patients do not fulfil the reimbursement criteria for PCSK9-i

**References for supplemental material**

1. Catapano AL, Graham I, De Backer G, et al. 2016 ESC/EAS Guidelines for the Management of Dyslipidaemias. Eur Heart J. 2016;37(39):2999-3058.
2. Overheid N. Bijlage 2 Regeling zorgverzekering 2019 [04-12-2019:[Available from: https://wetten.overheid.nl/jci1.3:c:BWBR0018715&bijlage=2&z=2019-12-04&g=2019-12-04.
3. Cannon CP, Blazing MA, Giugliano RP, et al. Ezetimibe Added to Statin Therapy after Acute Coronary Syndromes. N Engl J Med. 2015;372(25):2387-97.
4. Grundy SM, Stone NJ, Bailey AL, et al. 2018 AHA/ACC/AACVPR/AAPA/ABC/ACPM/ADA/AGS/APhA/ASPC/NLA/PCNA Guideline on the Management of Blood Cholesterol: Executive Summary: A Report of the American College of Cardiology/American Heart Association Task Force on Clinical Practice Guidelines. Circulation. 2019;139(25):e1046-e81.
