# Supplementary material for: Effects of a stepwise, structured LDL-C lowering strategy in patients post-acute coronary syndrome
Source: Neth Heart J. 2024 Jan 26;32(5):206–12. doi: 10.1007/s12471-023-01851-7 (PMC11039599; doi:10.1007/s12471-023-01851-7)
Supplement: Supplementary file 2 — Statistical analysis plan [file 12471_2023_1851_MOESM2_ESM.pdf]

DocuSign Envelope ID: 905090F3-A556-4E74-B507-3C4A5915E191

PENELOPE

Prevalence of unmet target LDL-C recommendations in very high risk patients despite high intensity lipid modifying therapy

Statistical Analysis Plan

v1.0 17FEB2021

Omar Khader A., Alings M.

DocuSign Envelope ID: 905090F3-A556-4E74-B507-3C4A5915E191

Statistical Analysis Plan

Version 1.0

Date: 17 Feb 2021

Written By:	*Signature:*	*Date:*
Aaram Omar Khader, co-principal Investigator PENELOPE	DocuSigned by: Aaram Omar Khader DFFE7B35A7B5466...	07-Dec-2022
Marco Alings, Principal Investigator PENELOPE	DocuSigned by: Marco Alings 7E7ED6405E33485...	17-nov-2022
Approved By: (WCN)	*Signature:*	*Date:*
Astrid Schut, Managing Director	DocuSigned by: Astrid Schut E6436C68EC9E446...	17-nov-2022

Synopsis

The primary objective of the PENELOPE study is to assess the prevalence of unmet target LDL-C (1.8 mMol/L) despite a standardized stepwise high intensity lipid modifying therapy in patients with ASCVD and at very high risk for recurrent events.

Consecutive patients presenting with a type I (N)STEMI and a history of ASCVD and/or Diabetes mellitus II will be included. Prevalence will be determined of unmet target LDL-C (1.8 mMol/L) at each therapeutic step (Baseline, high intensity statin therapy (HIST) , HIST+ ezetimibe, HIST + ezetimibe + PCSK9-I), and after one year of therapy.

Sample size calculation

The sample size is calculated to provide a prevalence (LDL>1.8 mMol/L in patients mentioned in the primary objective) with 95% confidence interval with a width (upper bound minus lower bound) of 5%.

The prevalence of LDL-C >1.8 mMol/L in patients treated with high-intensity statin and ezetimibe (~ primary objective) is estimated at 0.20 based on the IMPROVE-IT study. Seventy five percent of patients on simvastatin and ezetimibe in the IMPROVE-IT study have a LDL < 62 mg/dL (1.6 mMol/L). Since a higher threshold (1.8 mMol/L) and more potent statins are used in the current study, the prevalence is estimated at 20%. Using this assumption, 983 patients are needed to ensure a 95% confidence interval with a width of 5% (margin of error: 2.5%). The number is rounded up to 1000 patients.

$$\text{Sample size (n)} = \frac{Z^2 \times p \times (1-p)}{d^2}$$

Where Z= 1.96 (confidence interval 95 %), d= margin of error of 0.025 %, prevalence (p) = 0.2

$$\frac{1.96^2 \times 0.2 \times 0.8}{0.025^2} = 983$$

Primary Endpoint

prevalence of an LDL-C >1.8 mMol/L in a subgroup of very high risk patients, despite guideline based therapy with HIST and/or ezetimibe

Secondary Objective(s):

a) Prevalence of an LDL-C >1.8 mMol/L in patients with a medical history of ASCVD and/or T2DM and presenting with a new type I (N)STEMI, with or without lipid modifying therapy.
b) Prevalence of an LDL-C >1.8 mMol/L in patients as described in a.) after 4 weeks of HIST (i.e., atorvastatin ≥40 mg or rosuvastatin ≥ 20 mg; or the documented highest tolerated dose of a statin).
c) Prevalence of an LDL-C >1.8 mMol/L in patients as described in the primary objective after 4 weeks of HIST + ezetimibe 10 mg + alirocumab 75 Q2W or 150 mg Q2W.
d) Prevalence of an LDL-C >1.8 mMol/L after 1 year of follow up.
e) Adherence to lipid modifying therapy after 1 year
f) Prevalence of drug-related adverse events of the lipid modifying drugs and a subgroup analysis between male/ female and >70y and ≤70y.

g) Prevalence of discontinuation of the lipid modifying drugs and a subgroup analysis between male/ female and >70y and ≤70y.

h) Major adverse cardiovascular events (Myocardial infarction, stroke, (re-)hospitalization for cardiovascular diseases, revascularization and cardiovascular death) after 1 year

Statistical analysis

Statistical analysis will be descriptive only. The study is intended nor powered to perform intergroup analysis.
The prevalence of the patients not reaching the target level at baseline will be calculated by dividing the proportion of patients with LDL-C > 1.8 mMol/L at baseline by the total included patients.
For each consecutive step the net and the cumulative effect will be calculated. The net effect is the percentage of patients who reach the target (LDL-C ≤ 1.8 mMol/L) in the corresponding step. The cumulative effect is the percentage of all patients who reach the target (LDL-C ≤ 1.8 mMol/L) in the previous and the current steps. The labelling of the groups and the calculation of the net and cumulative effect are shown in figure 2.
In patients with Ezetimibe monotherapy because of a statin intolerance the net effect is calculated in the same manner as the patients with HIST end ezetimibe. The calculations will be performed for the total population and separately for patients with a history of ASCVD and DMII.
The results of the calculations will be presented in table 3.
The therapy during the first 12 weeks and the changes at one year follow-up will be presented in figure 3.
The cumulative effect in the first 12 weeks and at one year follow-up will be presented in figure 4.
The prevalence of drug-related adverse events and discontinuation rate of the lipid modifying drugs for the study population and subgroups (men and women, >70y and ≤70y) will be presented in a table. The intergroup analysis is performed using the Chi-squared test.

Prevalence of MACE is calculated as follows : $\frac{\text{Patients with MACE at T52}}{\text{Total patients at T52}}$ The same calculation will be used for patients with decreased adherence for each treatment group (HIST, HIST+ezetimibe, HIST + Ezetimibe + PCSK-9i) and will be presented in table 3.
In the above mentioned analysis an LCL-c threshold of 1.8 mmol/l is used, the same analysis will be carried out using an LDL-c threshold of 1.4 mmol/L.
The percentage of patients with an LDL >1.8 mmol/L and ≤ 2.6 mmol/L where a treatment with Alirocumab is started, will be calculated as follows:

$$\frac{\text{Patients with an LDL} > 1.8\ \text{mMol/L} \leq 2.6\ \text{mMol/L Alirocumab with}}{\text{Patients with an LDL} > 1.8\ \text{mMol/L} \leq 2.6\text{mMol/L on T8}}$$

DocuSign Envelope ID: 905090F3-A556-4E74-B507-3C4A5915E191

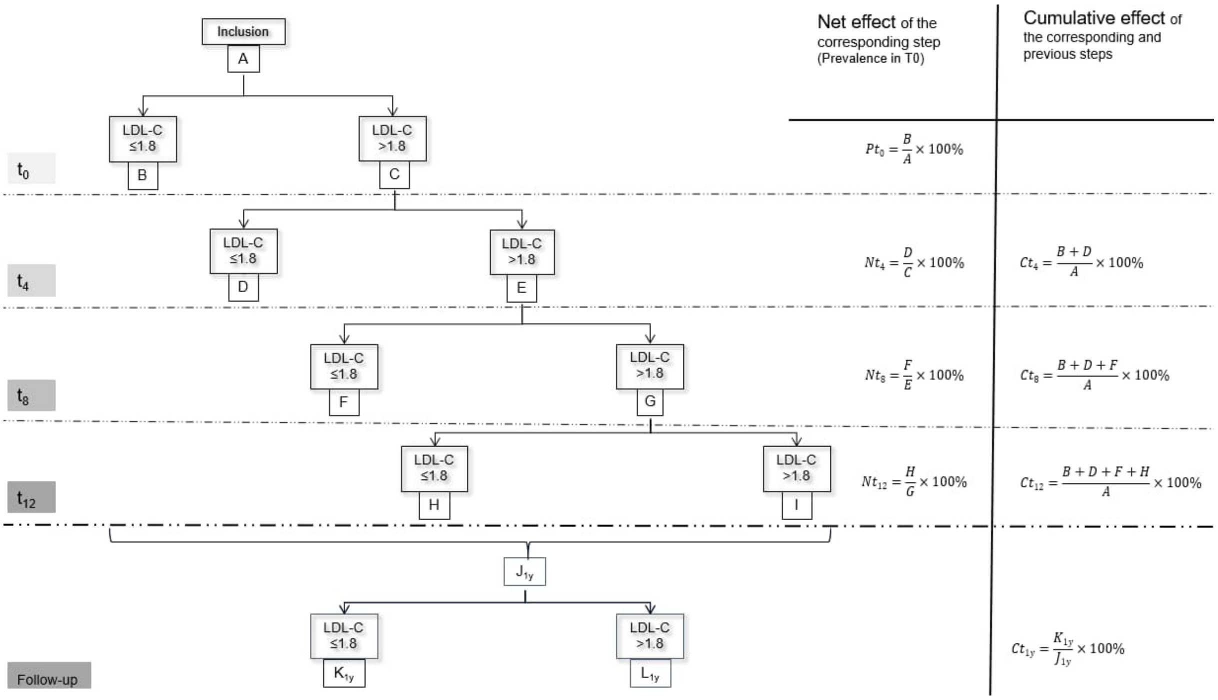

Figure 2 Statistical analysis

On the left side: Different groups are labelled (A,B,C,…) based on the LDL-value and the different stages/steps.
On the right side: The calculation of the net (Nt_x) and cumulative (Ct_x) effect for each step (T4,T8,T12) effect are shown. The net effect is the percentage of patients who reach the target (LDL-C ≤ 1.8 mMol/L) in the corresponding step. The cumulative effect is the percentage of all patients who reach the target (LDL-C ≤ 1.8 mMol/L) in the previous and the current steps
For T_0 the of calculation of the prevalence (Pt_0) of patients with an LDL ≤1.8mMol/L is shown.

Table 3 Summary of the collected data

	Net effect of each consecutive step			
	Pt_0	Nt_4	Nt_8	Nt_{12}
LDL≤ 1.8 mMol/L	*n (%)*	*n (%)*	*n (%)*	*n (%)*
	Cumulative effect of the consecutive steps			
	Pt_0	Ct_4	Ct_8	Ct_{12}
LDL≤ 1.8 mMo/Ll	*n (%)*	*n (%)*	*n (%)*	*n (%)*
	Prevalence of patients not reaching target at the different steps			
	T_0	T_4	T_8	T_{12}
LDL> 1.8 mMol/L	*n (%)*	*n (%)*	*n (%)*	*n (%)*
	One year follow up			
	All patients	*HIST*	*HIST + ezetimibe*	*HIST + Ezetimibe + PCSK-9i*
LDL≤ 1.8 mMo/Ll	*n (%)*	*n (%)*	*n (%)*	*n (%)*
↓ Adherence	*n (%)*	*n (%)*	*n (%)*	*n (%)*
MACE	*n (%)*	*n (%)*	*n (%)*	*n (%)*

HIST: High intensity statin therapy

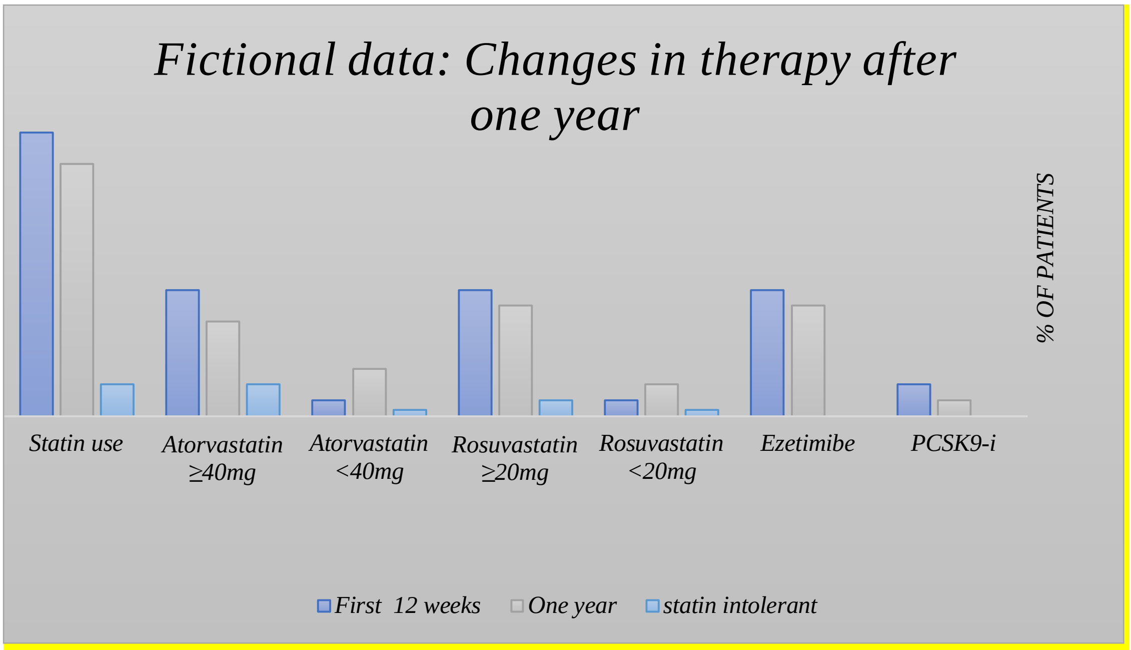

Figure 3 Changes in therapy after one year

This figure is for illustration purposes only. The data of this illustration is fictional.

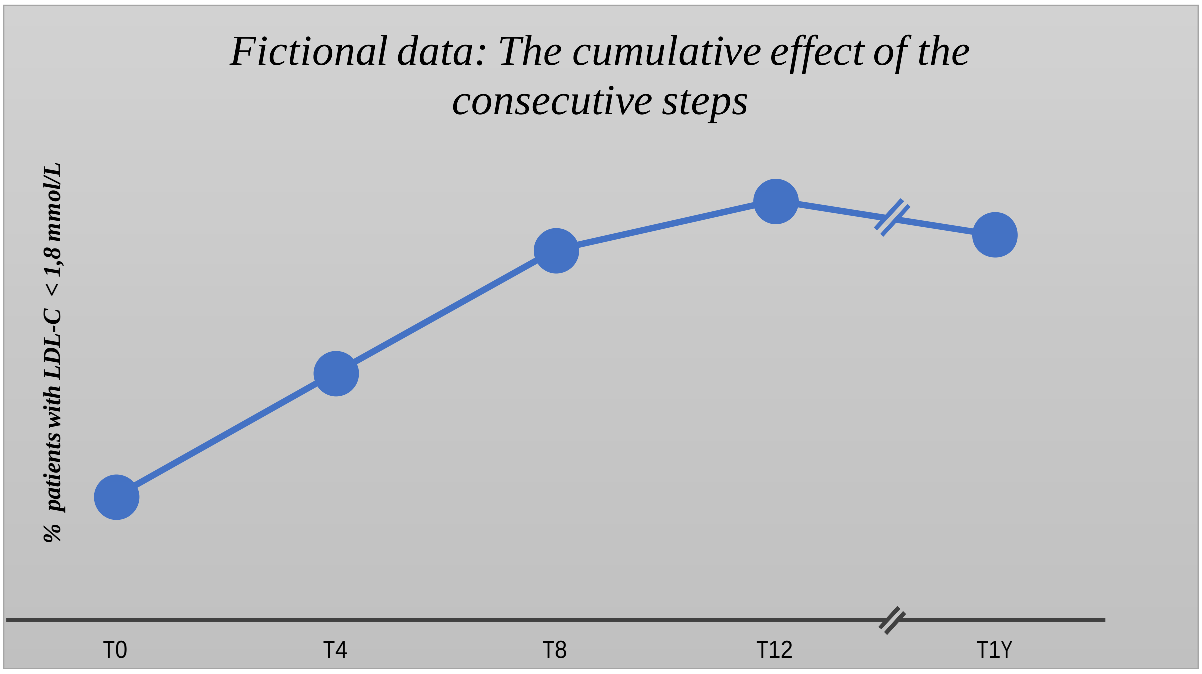

Figure 4 The cumulative effect of the consecutive steps

This figure is for illustration purposes only. The data of this illustration is fictional.

Study parameters collected:

T0:

- Medical history: ASCVD; T2DM; intolerances to alirocumab, ezetimibe or statins

-LDL-c

-Statin use at baseline

-ezetimibe use at baseline

T4:

-Final statin use at T4

-LDL-c

-Development of statin intolerance

-If statin intolerant:

-Which 3 statins are used

-Recurrent intolerance?

T8:

-Final statin use at t8

-LDL-c

DocuSign Envelope ID: 905090F3-A556-4E74-B507-3C4A5915E191

-Intolerance to Ezetimibe

-Which Alirocumab is started

T12:

-LDL-C

T52:

-LDL-c

-Statin use

-Alicrocumab use

-Adherence

-MACE

Additional parameters:

- CHF, hypertension, age (≥75 yr), diabetes, prior stroke, prior CABG, peripheral artery disease, eGFR <60, smoking [15]

-SAE and AE at each consecutive step

- Optional: non-HDL, triglyceride, plasma levels at each consecutive step

- ASALT, ALAT, AF, Bilirubine, Creatinin kinase, GGT, LVEF, pregnancy, alteration in non-study medication.

Data handling conventions:

The data is collected in electronic case report forms (castoredc.com). The data is exported in an Excel file and analysed using Python.

Data Handling Conventions For Patient Data

Out of range data:

LDL-c <0.5 mmol/L and > 7.5mmol/L are double checked in the patients' medical record.

The following data will be considered as missing values in the calculation:

LDL-c > 20 mmol/L

Missing data:

Missing values are handled using pairwise deletion. The case is excluded from the analysis if one or more data points needed for that particular calculation is missing.
